# Utilizing Autologous Multipotent Mesenchymal Stromal Cells and **β**-Tricalcium Phosphate Scaffold in Human Bone Defects: A Prospective, Controlled Feasibility Trial

**DOI:** 10.1155/2016/2076061

**Published:** 2016-04-07

**Authors:** Pavel Šponer, Stanislav Filip, Tomáš Kučera, Jindra Brtková, Karel Urban, Vladimír Palička, Zuzana Kočí, Michael Syka, Aleš Bezrouk, Eva Syková

**Affiliations:** ^1^Department of Orthopaedic Surgery, Charles University in Prague, Faculty of Medicine in Hradec Králové, Šimkova 870, 500 38 Hradec Králové, Czech Republic; ^2^Department of Oncology and Radiotherapy, Charles University in Prague, Faculty of Medicine in Hradec Králové, Šimkova 870, 500 38 Hradec Králové, Czech Republic; ^3^Department of Orthopaedic Surgery, University Hospital in Hradec Králové, Sokolská 581, 500 05 Hradec Králové, Czech Republic; ^4^Department of Radiology, University Hospital in Hradec Králové, Sokolská 581, 500 05 Hradec Králové, Czech Republic; ^5^Institute for Clinical Biochemistry and Diagnostics, Charles University in Prague, Faculty of Medicine in Hradec Králové, Šimkova 870, 500 38 Hradec Králové, Czech Republic; ^6^Institute of Experimental Medicine, Academy of Sciences of the Czech Republic, Vídeňská 1083, 142 20 Prague, Czech Republic; ^7^Department of Neuroscience, 2nd Faculty of Medicine, Charles University in Prague, V Úvalu 84, 150 06 Prague, Czech Republic; ^8^Department of Medical Biophysics, Charles University in Prague, Faculty of Medicine in Hradec Králové, Šimkova 870, 500 38 Hradec Králové, Czech Republic

## Abstract

The purpose of this prospective controlled study was to compare healing quality following the implantation of ultraporous *β*-tricalcium phosphate, containing either expanded autologous mesenchymal stromal cells (trial group, 9 patients) or *β*-tricalcium phosphate alone (control group, 9 patients), into femoral defects during revision total hip arthroplasty. Both groups were assessed using the Harris Hip Score, radiography, and DEXA scanning at 6 weeks and 3, 6, and 12 months postoperatively. A significant difference in the bone defect healing was observed between both groups of patients (*P* < 0.05). In the trial group, trabecular remodeling was found in all nine patients and in the control group, in 1 patient only. Whereas, over the 12-month follow-up period, no significant difference was observed between both groups of patients in terms of the resorption of *β*-tricalcium phosphate, the significant differences were documented in the presence of radiolucency and bone trabeculation through the defect (*P* < 0.05). Using autologous mesenchymal stromal cells combined with a *β*-tricalcium phosphate scaffold is a feasible, safe, and effective approach for management of bone defects with compromised microenvironment. The clinical trial was registered at the EU Clinical Trials Register before patient recruitment has begun (EudraCT number 2012-005599-33).

## 1. Introduction

Bone defects subsequent to trauma, tumor resection, infection, and prosthesis loosening pose an important clinical problem. The character of bone defects is classed as either cavitary or segmental. A cavitary defect is a contained lesion and represents an excavation of the cancellous or endosteal cortical bone with no violation of the outer cortical shell. A segmental defect is characterized by the loss of bone in the supporting cortical shell [1]. The ability of bone to regenerate may be compromised by the character, the location, and the size of the bone defect [2]. Conventional treatment to augment bone healing is based on cancellous bone autografts, morselized allografts, and bone graft substitutes application [3].

Based on the results of preclinical studies, hydroxyapatite and calcium phosphate are ideal substrates for use as a matrix due to their excellent osteoconductive properties [4–6]. Usually, when using hydroxyapatite, it is combined with either *β*-tricalcium phosphate, resulting in biphasic calcium phosphate, or biodegradable polymers, resulting in composite biomaterials.

Recently, initial clinical reports have investigated the potential of expanded MSCs in the repair of long bone defects [7, 8]. Though the preliminary results are promising, the development of cell-based strategies still faces two major tasks, including the optimization of the techniques used for cell cultivation and the characterization of the scaffold used as a cell carrier.

To begin addressing these tasks, we report the first prospective, controlled clinical trial utilizing expanded autologous MSCs for the regeneration of bone defects. This study compares the quality of healing following the implantation of ultraporous *β*-tricalcium phosphate synthetic graft material (Vitoss®, Stryker, Kalamazoo, USA) serving as a carrier of either expanded autologous MSCs (suspension of autologous MSC 3P in 1.5 mL, Bioinova, Ltd., Prague, Czech Republic) or *β*-tricalcium phosphate only into femoral defects during revision total hip arthroplasty. The newly used methodology presented in this study could be applied in many other cases of orthopaedic surgery and traumatology.

## 2. Material and Methods

### 2.1. Patients

Following State Institute for Drug Control of the Czech Republic (authorization # 12/2012; identification number 195432/12-I) and the Local Ethics Committee (authorization # 12/2012) approval, 18 patients requiring a femoral revision were recruited to participate in this phase IIa trial (protocol AMSC-BDT-001), EudraCT number 2012-005599-33 (authorization # 12/2012). No power analysis was performed to define the sample size, but instead the number of participants for this trial was chosen based on feasibility rather than statistical exactness as a first-in-human investigation. Male and female participants with an established diagnosis of aseptic loosening of a total hip arthroplasty (bone defect of the proximal femur seen on plain radiographs, ASA score from I to III for subjects aged 18 to 65 years or ASA score from I to II for subjects aged 66 to 75 years) were included if they were able and willing to read, understand, and sign an informed consent form. Patients were excluded from participation if they had or exhibited one of the following conditions: cancer, pelvic radionecrosis, rheumatoid arthritis, steroid or immunosuppressive therapy, malnutrition, active infectious disease, previous infection at the site of total hip arthroplasty, pregnancy, alcohol or drug abuse, and any significant medical condition that would compromise the condition of the patient (recent myocardial infarction, congestive heart failure, kidney, or liver failure). The average age of the patients at the time of operation was 67 (59–72) years in the trial group and 70 (65–75) years in the control group, respectively (Tables 1(a) and 1(b)).

### 2.2. Study Design

**Figure 1:**
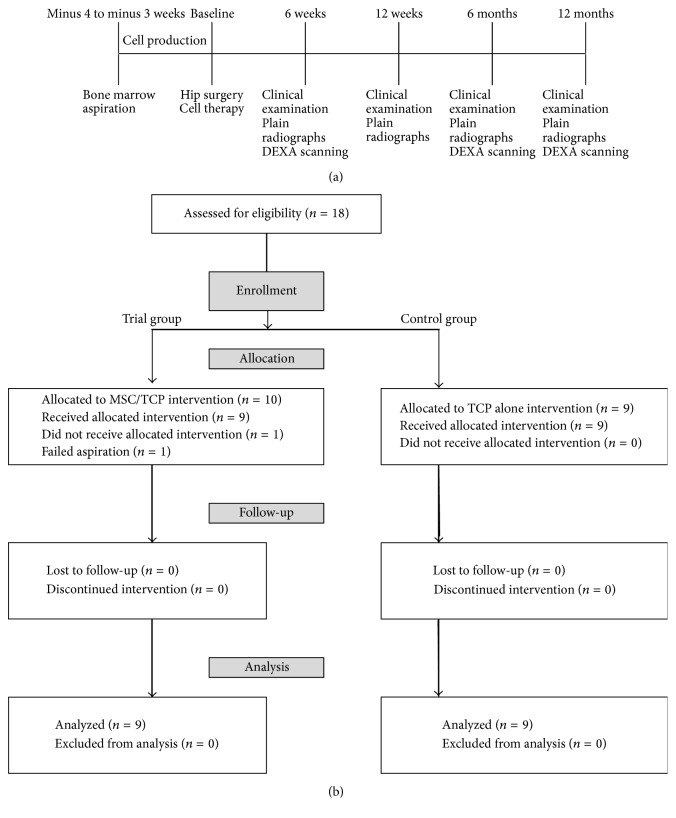
(a) Study timeline. (b) Participant distribution.

From January 2013 to May 2015, 18 osseous defects of 18 patients were treated and evaluated for bone regeneration. Half of the subjects received a synthetic graft material serving as a carrier of expanded autologous MSCs, and the other half received the synthetic graft material only. Due to the nature of the trial group requiring autologous bone marrow aspiration, surgeon and patient masking was not possible. However, evaluation of treatment was done independently by examiners of primary outcome measures (radiographic and DEXA scanning assessment).  Figure 1(a) shows the trial timeline, and Figure 1(b) displays the consort diagram of the patient allocation to the different groups.

### 2.3. Clinical Procedures

**Figure 2:**
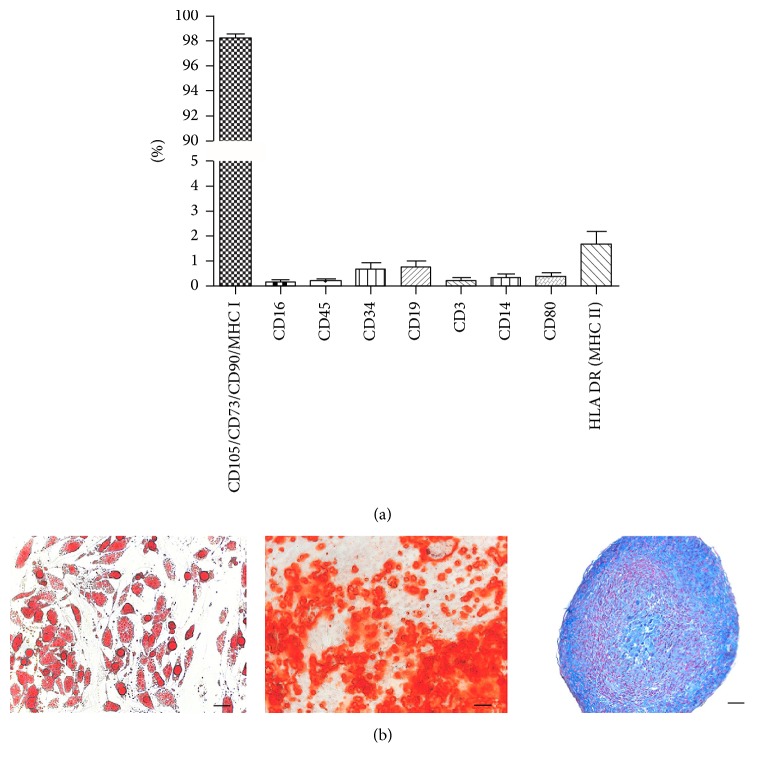
Administered product, suspension of autologous MSC 3P in 1.5 mL (Bioinova, Ltd., Prague, Czech Republic), was analyzed for the expression of MSC surface markers and surface markers of possible contaminating cells (a) and tested for multipotent differentiation capacity (b).* In vitro* differentiation into adipocytes ((b) left, scale bar 100 *μ*m), osteoblasts ((b) middle, scale bar 100 *μ*m), and chondrocytes ((b) right, scale bar 50 *μ*m).

The good manufacturing practice production of MSCs has been previously described [6]. Following State Institute for Drug Control and the Local Ethics Committee approval, nine human participants, having given informed consent, underwent a bone marrow aspiration of the anterior ilium under local anesthetic (EudraCT number 2012-005599-33). Collected marrow (10–12 mL) was transferred to a sterile collection kit (Bioinova, Ltd., Prague, Czech Republic) and transported to a GMP facility (Bioinova, Ltd.) in order to be further processed. Briefly, bone marrow was applied on Gelofusine® (B. Braun, Melsungen AG, Germany) and mononuclear fraction was collected and used for cultivation. Mononuclear fraction was seeded on plastic flasks (70000–140000 cells/cm^2^, TPP Techno Plastic Products AG, Trasadingen, Switzerland) and allowed to adhere. Nonadherent cells were removed after 24–48 hours by replacing the media. Adherent cells were cultured at 37°C in a humidified atmosphere containing 5% CO_2_ in enriched MEM Alpha (Lonza, Walkersville, Maryland, USA) media containing platelet lysate (5%; Bioinova, Ltd.) and gentamicin (10 *μ*g/mL; Gentamicin Lek®; Lek Pharmaceuticals, Ljubljana, Slovenia). The media were changed twice a week. According to their spindle-shaped morphology and plastic adherence, the cells were identified as MSCs. After reaching near-confluency, cells were harvested by a TrypLE*™* (Life Technologies, Carlsbad, California, USA), passaged, and seeded again onto a fresh plastic surface. Cells were harvested during the 3rd passage, 4 weeks after the initial seeding. Cells were counted and analyzed for surface markers (Figure 2(a)), microbiological contamination, and sterility. Several benches of the product were tested for MSC multilineage potential to differentiate into adipogenic, osteogenic, and chondrogenic cell linages (Figure 2(b)). The cell product suspension of autologous MSC 3P in 1.5 mL containing 15 ± 4,5 × 10^6^ of autologous MSCs was then released to be used for individual revision hip surgery.

For the 18 patients selected to participate in this study, the revision hip procedure was done using a Bauer approach. After removal of the loosened component of the previously implanted hip replacement and careful curettage of the scar and granulation tissue from the prosthetic bed, the revision prosthesis was anchored into its definitive position and the size of the bone defect was measured. In the trial group (*n* = 9), 1.5 mL of the MSC suspension (15 ± 4,5 × 10^6^cells) was applied to an absorbable sponge (Vitoss, Stryker, Kalamazoo, USA), 5 cc soaked in 3.5 mL autologous blood. The sponge was then delivered into the femoral defect and the wound was closed. In the control group (*n* = 9), the absorbable sponge (Vitoss, Stryker, Kalamazoo, USA) only, 5 cc soaked in 3.5 mL autologous blood and 1.5 mL sterile saline, was implanted into the bone defect. Antibiotic prophylaxis lasted 24 hours. Low-molecular-weight heparin was used to prevent thromboembolic disease during the following 5 weeks. Postoperative physiotherapy started the first post-op day; full weight-bearing was allowed after 12 to 24 weeks.

### 2.4. Outcome Assessments

The patients were clinically examined 1 day prior to the operation and 6 weeks and 3, 6, and 12 months postoperatively. The minimal follow-up was 12 months; all patients are still being followed. The Harris Hip Score was calculated to evaluate pain and function [9].

The radiographs were taken using standard projections and evaluated independently by three reviewers. The data were than checked for interobserver agreement. In the case of differences, the patient's radiograph was reevaluated by all three observers together. Loss of bone stock was correlated using the preoperative radiographs and by assessing perioperatively found defects. The classification system of the American Academy of Orthopaedic Surgeons was used [10]. The presence of bone graft material within soft tissue, rim radiolucency surrounding the grafted defect, resorption of graft material, and bone trabeculation through the defect were evaluated at each time point according to Anker et al. [11]. In the final follow-up, radiographic classification of the bone defect healing was performed using the guidelines of Gie, distinguishing between the following four main findings [12]:

1. No change: completely unchanged bone graft material findings, compared to the first postoperative radiograph.
2. Trabecular incorporation: any change in the structure of the bone graft material from the postoperative radiograph without any distinctive orientation.
3. Trabecular remodeling: the bone graft material which has changed into a pattern of trabeculae running from the endosteal cortex into the implant along the supposed lines of stress.
4. Cortical healing: postoperative thickening of the cortex compared to a preoperatively thinned-out cortex or a cortex with localized endosteal erosions. Consequently, if the cortex is not interfered by the loosening process, the term cortical healing is not applicable.

Combinations of findings were possible; cortical healing described only the reactions in the cortical bone, whereas trabecular incorporation and remodeling relate to the space between the endosteum and implanted endoprosthesis. The extent of heterotopic ossification was graded using the classification of Brooker et al. [13].

DEXA scanning was performed on each patient preoperatively as well as at 6 weeks and 6 and 12 months postoperatively. A Discovery A scanner (Hologic, Bedford, MA) was used to obtain scans of each patient in a supine position with the leg held in a neutral position, toes to the ceiling, and the foot strapped into a rigid holder in order to reduce rotational variation between scans. The periprosthetic femoral bone mineral density (BMD) was determined for regions of interest corresponding with the filled area within the proximal femur, using the software version 13.3.0.1:3 for the Discovery A. We analyzed three postoperative scans for each patient and calculated the BMD for the selected region of interest, which was then converted to a percentage of the first postoperative value.

### 2.5. Statistical Analysis

The Mann-Whitney test was used to determine differences between the presence of a rim radiolucency surrounding the grafted defect, resorption of graft material, and bone trabeculation through the defect for both trial and control groups, to determine differences between the radiographic bone defect healing and bone density within the filled area. A value of *P* < 0.05 was considered to be significant.

## 3. Results

### 3.1. Study Design and Patients

Throughout the study, there were no serious, study-related adverse events that were reported in examination of comprehensive safety assessments during the trial. It should be noted that, in order to obtain a total of 9 patients in the trial group, it required a total of 10 participants to be included in this group because bone marrow could not be processed from one patient due to aspirate clotting and it required one repeated aspiration due to aspirate clotting too.

### 3.2. Clinical Results

To date, none of the implants required rerevision. In the control group, the mean Harris Hip Score improved from 49 points to 83 points and the average pain score improved by 22 points to 43 points, postoperatively. In the trial group, the mean Harris Hip Score improved from 51 points to 86 points and the average pain score improved by 23 points to 44 points, postoperatively (Tables 2(a) and 2(b)).

### 3.3. Radiography

In the final follow-up session, a significant difference in the bone defect healing was observed between both groups of patients (*P* < 0.05). In the trial group, 2 patients had a cortical defect in zone 1 and zone 7, respectively, of which both showed cortical repair. In the filled medullary cavity, there was trabecular remodeling in 9 patients. None of the 9 implants showed any signs of subsidence. In contrast to this, in the control group, one patient had a cortical defect in zone 6, of which cortical repair was observed. In the filled medullary cavity, there was trabecular remodeling in 1 patient only, trabecular incorporation in 4 patients, and no change in 4 patients. One implant revealed signs of initial vertical instability not affecting its osteointegration. With the numbers available, statistical analysis showed no relationship between age and bone defect healing.

**Figure 3:**
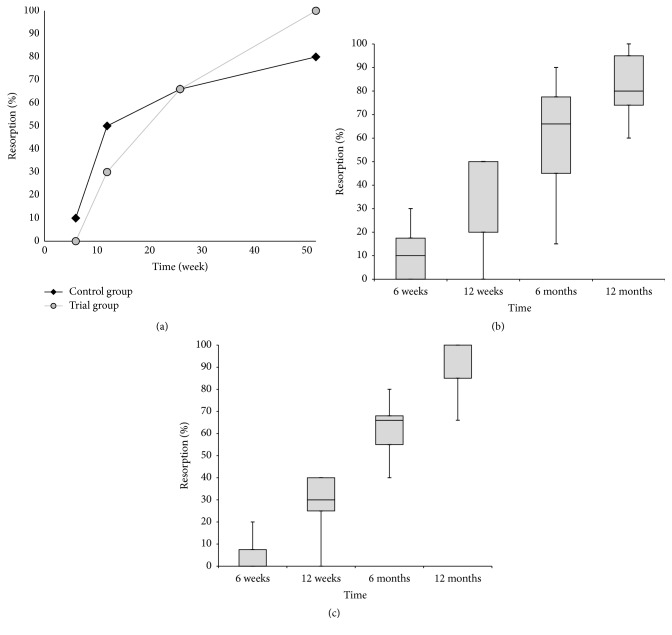
(a) The progress of resorption of the graft material over time. The rate of resorption is expressed as the median percentage of the initial filling visible on the postoperative radiograph. No significant difference was observed between both groups of patients. (b) The box plot graph showing resorption of the graft material in the control group. (c) The box plot graph showing resorption of the graft material in the trial group. The bottom and top of the box are the first and third quartiles, and the band inside the box is the median. The ends of the whiskers represent the minimum and maximum.

**Figure 4:**
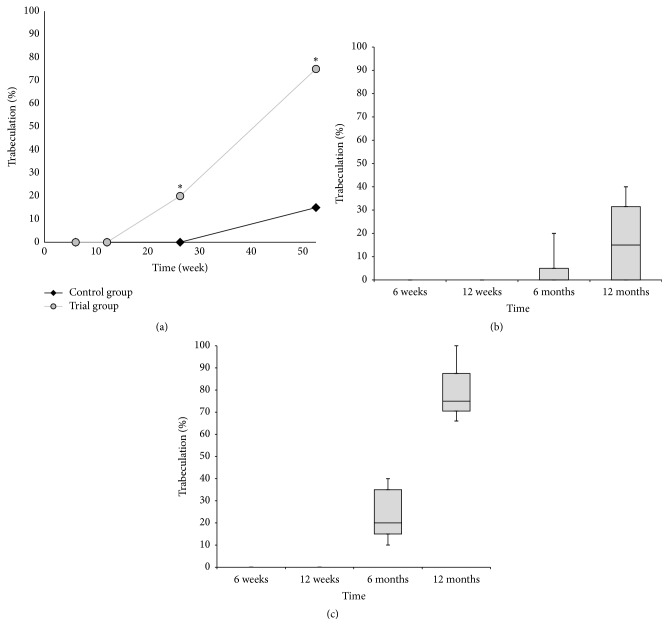
(a) The progress of bone trabeculation over time. The trabeculation is expressed as the median percentage of the initial filling visible on the postoperative radiograph. The asterisk (*∗*) indicates significant difference of the trial group in comparison to the control group. (b) The box plot graph showing bone trabeculation in the control group. (c) The box plot graph showing bone trabeculation in the trial group. The bottom and top of the box are the first and third quartiles, and the band inside the box is the median. The ends of the whiskers represent the minimum and maximum.

**Figure 5:**
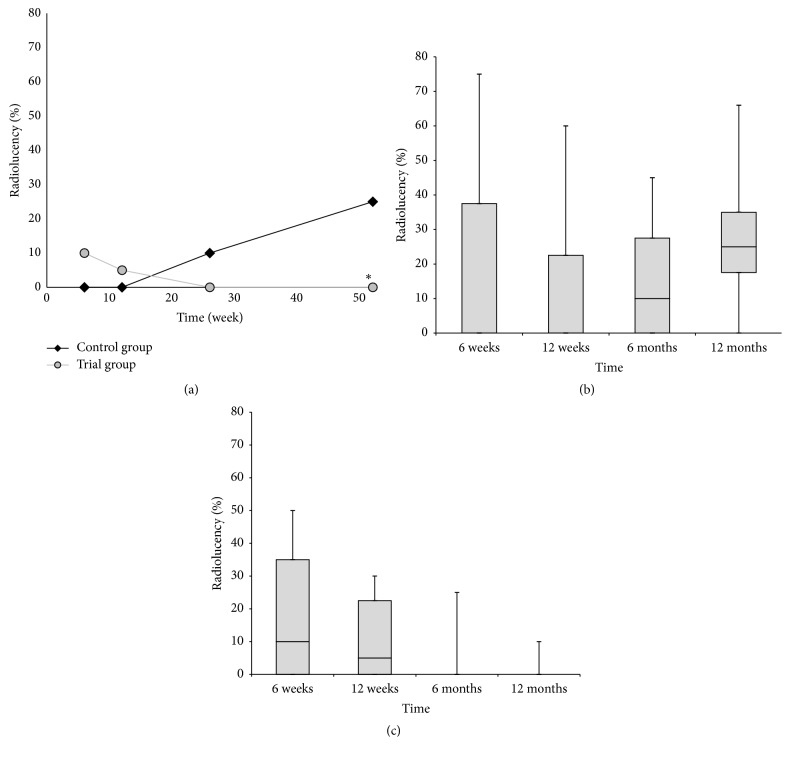
(a) The progress of radiolucency over time. The radiolucency is expressed as the median percentage of the initial filling visible on the postoperative radiograph. The asterisk (*∗*) indicates significant difference of the trial group in comparison to the control group. (b) The box plot graph showing the progress of radiolucency in the control group. (c) The box plot graph showing radiolucency in the trial group. The bottom and top of the box are the first and third quartiles, and the band inside the box is the median. The ends of the whiskers represent the minimum and maximum.

**Figure 6:**
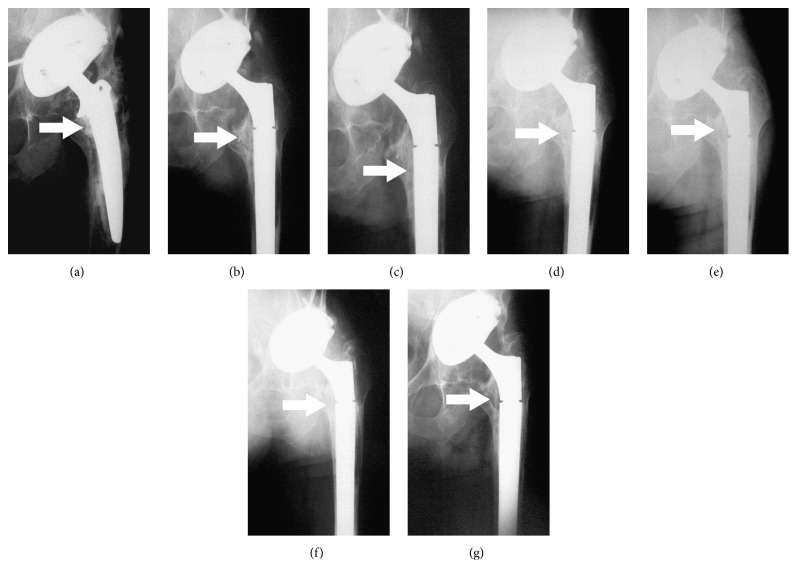
(a) Anteroposterior radiograph of a 68-year-old man (case number 6; trial group) with aseptic loosening of the femoral component which migrated into the varus position with contemporary formation of the medial bone defect of the femur; the femoral component is inside the bone defect. (b) Complete filling of the bone defect with tricalcium phosphate synthetic graft material combined with expanded multipotent mesenchymal stromal cells (MSCs) immediately after surgery. (c) Disappearing rim radiolucency surrounding the grafted defect distomedially at 6 weeks after surgery. (d) Initial peripheral resorption of the graft material combined with expanded MSCs at 12 weeks after surgery. (e) Gradual resorption within the grafted defect at 6 months after surgery. (f) Peripheral bone trabeculation through the defect at 9 months after surgery. (g) Complete resorption of tricalcium phosphate synthetic graft material with trabecular remodeling at 12 months after surgery. All described bone changes are marked by an arrow.

**Figure 7:**
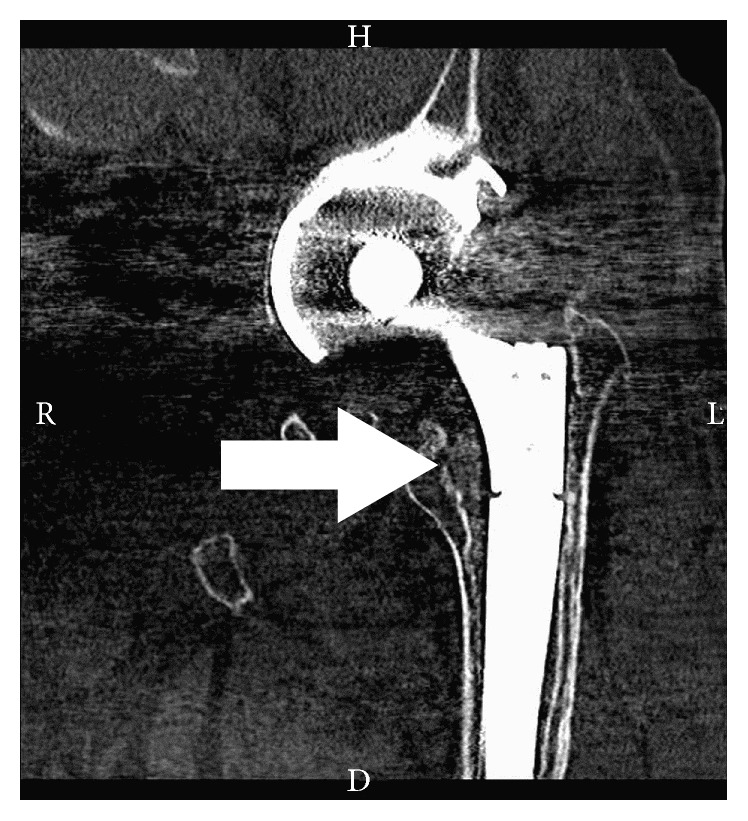
Corresponding coronal CT scan obtained for the same patient shown in previous figures (case number 6; trial group) at 12 months after surgery; the defect was filled with tricalcium phosphate synthetic graft material combined with expanded multipotent mesenchymal stromal cells (MSCs). The medial femoral bone defect is nearly completely filled with newly formed bone tissue (marked by an arrow).

Whereas, over the 12-month follow-up period, no significant difference was observed between both groups of patients in terms of the resorption of *β*-tricalcium phosphate, the significant differences were documented between trial and control groups in the presence of radiolucency and bone trabeculation through the defect (*P* < 0.05). Resorption of the graft material increased over the first 12 months, from close to zero at 6 weeks to between 80% and 100% at the end of the 12-month follow-up period (Figures 3(a)–3(c)). Trabeculation increased steadily with time for all defects filled with the MSC suspension placed onto *β*-tricalcium phosphate. In contrast, bone trabeculation in defects filled with *β*-tricalcium phosphate alone was significantly slower and less extensive (*P* < 0.05) (Figures 4(a)–4(c)). Whereas the value for the presence of a radiolucency decreased in the trial group, from an average of approximately 10% at 6 weeks to zero by the end of the follow-up period, in the control group, the value for the presence of radiolucency within the defect increased steadily from zero at 12 weeks to 25% on average by 12 months (*P* < 0.05) (Figures 5(a)–5(c)). The bone defect healing course in the trial group using a plain radiograph is seen in Figures 6(a)–6(g), with a supplementary 12-month CT scan in Figure 7.

### 3.4. DEXA Scanning

**Figure 8:**
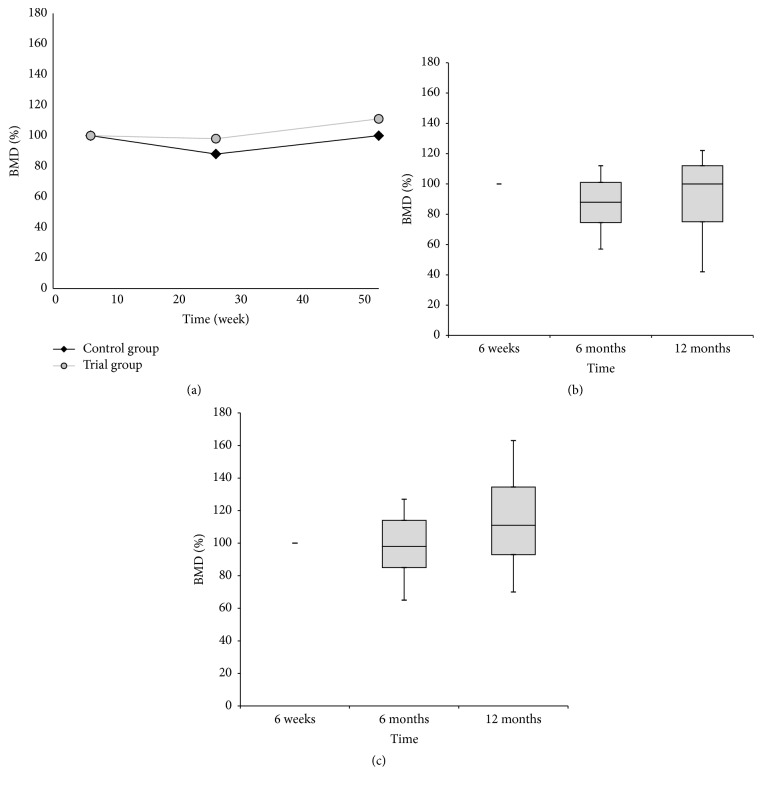
(a) The progress of DEXA bone mineral density (BMD) values in the trial and control group over time. The BMD value is expressed as the median percentage of the BMD values obtained from the first postoperative scan at 6 weeks. No significant difference was observed between both groups of patients. (b) The box plot graph showing BMD values in the control group. (c) The box plot graph showing BMD values in the trial group. The bottom and top of the box are the first and third quartiles, and the band inside the box is the median. The ends of the whiskers represent the minimum and maximum.

All 18 patients underwent their preoperative, 6-week, 6-month, and 12-month postoperative scans, but only postoperative scans were used for evaluation because of the presence of the original loosened femoral component in the region of interest in some cases. There were no complications related to the scanning. At 6 months after operation, there was a mild decrease in median BMD to 98% in the trial group with an increase in median BMD to 111% at 12 months after surgery. In the control group, there was a marked decrease in median BMD to 88% at 6 months after surgery with a subsequent increase of median BMD to 100% at 12 months after operation. These changes were not statistically significant (Figure 8).

### 3.5. Complications

In the trial group, 1 patient underwent a closed reduction of a prosthesis dislocated 4 months following the surgery. Other types of complications included pulmonary embolism 10 weeks following surgery in 1 patient. In the control group, 1 patient underwent open reduction for a prosthesis dislocated 5 months after operation. Other types of complications included intraoperative arrhythmia in 1 patient.

## 4. Discussion

An extensive body of preclinical data on MSC therapy has been accumulated and thus MSC therapy is entering a new era, shifting the focus from initial studies on feasibility to the optimization of therapeutic efficacy so as to achieve more consistent and sustained clinical potency. Bone tissue engineering has been one of the most promising areas of research, providing a potential clinical application to cure bone defects. Recently, various stem cells, including embryonic stem cells, MSCs from bone marrow, umbilical cord blood-derived mesenchymal stem cells, adipose tissue-derived stem cells, muscle-derived stem cells, and dental pulp stem cells, have received extensive attention in the field of bone tissue engineering due to their distinct biological capability to differentiate into osteogenic lineages.

Some authors have reported that the application of stem cells to bone tissue engineering requires inducing the* in vitro* differentiation of these cells into bone forming cells, osteoblasts, for which efficient, well-defined, and proficient* in vitro* differentiation protocols are needed [14]. This would reduce the likelihood of spontaneous differentiation into divergent lineages and increase the available cell source for application to bone tissue engineering therapies [14].

MSCs readily undergo osteogenesis upon treatment with dexamethasone or bone morphogenetic proteins (BMPs)* in vitro*, suggesting that they may serve as long-term precursors for bone regeneration [15]. The propensity of MSCs to bone tissue* in vivo* has also been indicated by two MSC cardiac therapy studies demonstrating unexpected myocardial bone tissue formation or calcification upon cell transplantation [16, 17].

However, although MSCs exhibit prominent multilineage differentiation potential, recent studies have revealed that this cellular feature contributes little to their therapeutic benefits [18]. It is widely accepted that the therapeutic potential of MSCs is derived from their secretion of a variety of growth factors and cytokines [19, 20].

There is a need for therapy of bone defects throughout the whole spectrum of orthopaedic surgery to replace bone substance which has been lost due to congenital disorders, trauma, and inflammatory events, for reconstructive surgical procedures in hip arthroplasty revision surgery and surgery for bone tumors. While a well-vascularized host bed is present in cavitary defects after curettage of benign bone tumors, the circumstances of femoral revision dictate a compromised microenvironment with poor vascularization to neighboring implanted metallic endoprosthesis [21]. To evaluate the safety and efficacy, we compared the healing of bone defects after the application of* in vitro* expanded MSCs combined with ultraporous *β*-tricalcium phosphate synthetic graft material and *β*-tricalcium phosphate synthetic graft alone within 12-month follow-up period.

Regenerative medicine aims to use tissue engineering to restore damaged tissue. In this report, we describe the first human controlled trial employing stem cell therapy for the regeneration of long bones. Our comparative analyses of treatment sites are based on clinical, radiographic evaluation and DEXA scanning. In the trial and control groups, the presence of femoral component and cerclage strips has prevented us from using conventional computed tomography and magnetic resonance imaging because of metal-induced artifacts. Metal in an anatomic area of interest produces both a large area of signal void and extensive distortion around the implant [22]. In most instances, the signal void and distortion preclude acquisition of useful information in the immediate area of the metallic object. Compared to preclinical studies, a biopsy could not be done as a standard procedure in patients who did not have complaints due to ethical constraints. On the other hand, based on histological and radiographic observation in 6 postmortem femurs and 8 biopsies, radiographic changes have been reported to be representative of viable bone. Radiographically, cortical healing and trabecular remodeling correspond to viable bone [23]. In the trial group, radiolucent zones between *β*-tricalcium phosphate material combined with MSCs and the surrounding bone faded, new bone developed, and trabecular remodeling was observed in all 9 patients at 12 months after surgery. In contrast to this, in 4 patients of the control group, radiolucent zones developed within *β*-tricalcium phosphate filling at 12 months postoperatively because of biomaterial resorption without new bone formation.

This prospective, controlled study is limited by the small patient numbers and short duration of follow-up. Nevertheless the follow-up was sufficient to reveal a difference in the presence of a radiolucency and bone trabeculation between defects filled by MSCs combined with tricalcium phosphate and those filled by tricalcium phosphate alone. Also, the time of clinical and radiographic healing and revision total hip arthroplasty complication rate are comparable to those of recent surveys [24, 25]. Despite this weakness, the study is the first prospective radiographic analysis on tricalcium phosphate synthetic graft material combined with expanded MSCs.

## 5. Conclusions

The safety of using an ultraporous *β*-tricalcium phosphate synthetic graft material combined with expanded MSCs in bone defects with poor vascularization to neighboring implanted metallic endoprostheses is beyond doubt, and the first steps toward creating a microenvironment for true bone regeneration have been taken.

Our results show that using autologous MSCs combined with a *β*-tricalcium phosphate scaffold is a feasible, safe, and effective approach for management of bone defects with compromised microenvironment. In the trial group, there were no serious study-related adverse events and none of the implants required rerevision. Patients improved in all clinical assessments. We propose that this newly used methodology could be applied in many other cases of orthopaedic surgery and traumatology.

## Figures and Tables

**(a):** 

Case	*A*	*B*	*C*	*D*	*E*	*F*	*G*	*H*	*I*	*J*	*K*	*L*
1	2	70	81	34	2	0	1	0	1	II	13.5	0
2	1	61	87	26	3	3	2	1	0	II	19.1	0
3	1	59	86	28	3	0	1	0	1	III	13.5	1
4	2	71	60	25	2	0	1	2	1	III	4.9	0
5	2	72	72	32	2	0	1	3	1	III	11.3	1
6	1	68	86	28	2	1	1	0	1	III	13.2	0
7	1	67	102	32	1	1	1	0	1	III	6.0	1
8	1	68	80	25	1	0	1	2	1	II	50.0	0
9	2	70	73	30	2	0	1	2	1	II	16.0	1

**(b):** 

Case	*A*	*B*	*C*	*D*	*E*	*F*	*G*	*H*	*I*	*J*	*K*	*L*
1	1	68	94	31	1	0	1	0	1	II	13.5	1
2	2	68	87	31	1	0	1	1	0	II	18.75	0
3	2	66	86	33	1	0	1	2	1	II	15.0	0
4	2	75	54	20	1	0	1	0	1	II	22.5	0
5	2	70	93	32	1	1	3	1	2	II	12.0	0
6	2	70	70	27	2	1	1	2	1	II	24.0	0
7	2	69	83	31	2	0	1	0	1	II	10.5	1
8	2	75	78	29	1	1	1	0	1	IV	16.0	1
9	2	65	73	30	2	0	1	2	1	II	16.0	1

*A*: gender: 1: male; 2: female; *B*: age at revision (years); *C*: weight (kg); *D*: body mass index (kg/m^2^); *E*: primary diagnosis: 1: osteoarthritis, 2: hip dysplasia, and 3: osteonecrosis; *F*: number of previous revisions; *G*: revision indication: 1: aseptic loosening, 2: instability of arthroplasty, and 3: suspected septic loosening (negative cultures); *H*: cup revision: 0: none, 1: uncemented cup, 2: uncemented cup + morselized allogeneic bone graft, and 3: cemented cup + Burch Schneider + morselized allogeneic bone graft; *I*: femoral component removed: 0: well-fixed and positioned femoral component not removed, 1: cemented, and 2: cement spacer; *J*: AAOS defect classification: II: cavitary, III: combined segmental and cavitary, IV: malalignment, *K*: size of the defect (cm^3^); *L*: femoral reinforcement: 0: none and 1 cerclage strips.

**(a):** 

Case	*A*	*B*	*C*	*D*	*E*	*F*	*G*	*H*	*I*
1	12	67 + 24	20 + 24	1	0	0	1	1	3
2	12	86 + 14	44 + 0	1	0	0	1	1	3
3	12	41 + 50	10 + 34	1	0	0	1	1	3
4	12	32 + 52	10 + 34	1	0	0	3	2	3, 4
5	12	27 + 63	10 + 34	2	1	0	1	1	3, 4
6	12	45 + 30	20 + 24	2	0	1	4	1	3
7	12	40 + 55	20 + 20	1	0	0	1	1	3
8	12	83 + 7	40 + 4	1	0	0	1	1	3
9	12	34 + 44	10 + 34	2	0	0	0	1	3

**(b):** 

Case	*A*	*B*	*C*	*D*	*E*	*F*	*G*	*H*	*I*
1	12	52 + 30	30 + 14	1	0	0	0	0	1
2	12	52 + 19	20 + 24	2	0	0	0	0	1
3	12	58 + 36	20 + 24	3	1	0	0	0	3
4	12	49 + 38	20 + 24	1	0	0	0	1	2
5	12	41 + 39	20 + 20	2	0	0	0	0	2
6	12	44 + 34	20 + 20	1	0	2	0	0	2
7	12	41 + 35	20 + 24	2	0	0	0	0	2
8	12	44 + 38	20 + 24	1	0	0	3	3	1, 4
9	12	60 + 34	20 + 24	1	0	0	0	0	1

*A*: follow-up (months); *B*: preoperative Harris Hip Score ± postoperative (points); *C*: preoperative pain score ± the postoperative increase (points); *D*: Trendelenburg sign: 1: preoperative positive and postoperative negative, 2: preoperative positive and postoperative positive, and 3: preoperative negative and postoperative negative; *E*: orthopaedic complications: 0: none and 1: dislocation; *F*: other complications: 0: none, 1: pulmonary embolism, and 2: cardiac arrhythmia; *G*: heterotopic ossification preoperatively: 0: none, 1: grade I, 3: grade III, and 4: grade IV; *H*: heterotopic ossification at latest followup: 0: none, 1: grade I, 2: grade II, and 3: grade III; *I*: radiographic result: 1: no change, 2: trabecular incorporation, 3: trabecular remodeling, and 4: cortical healing.
